# Sporadic Intra-Abdominal Desmoid: A Rare Presentation as a Hepatic Mass

**DOI:** 10.1155/2012/245671

**Published:** 2012-10-04

**Authors:** Shahana Gupta, Udipta Ray, Souvik Chatterjee, Sanjeev Kumar, Ayusman Satapathy, Shamita Chatterjee, Tamal Kanti Choudhury

**Affiliations:** Department of Surgery, Medical College, Kolkata, 88, College Street, Kolkata 700073, India

## Abstract

We report an unusual presentation of a sporadic intra-abdominal desmoid tumour, possibly arising from the diaphragm, masquerading as a hepatic mass in a young female without any history of surgery or trauma. Histopathology ruled out a hepatic origin of the tumour as was inferred from pre- and intraoperative evaluation. Immunohistochemistry showed positivity of lesional fibroblastic cells for **β**-catenin and negativity for CD34, CD117, EMA, SMA, desmin, vimentin, cytokeratin, and ALK1 thereby confirming the diagnosis of a desmoid tumour. There exist only a few reports in the literature on desmoids related to the diaphragm, but only one on a diaphragmatic desmoid that is possibly primary.

## 1. Introduction

Desmoid tumours are monoclonal, fibroblastic proliferations arising from musculoaponeurotic structures. These lesions infiltrate locally, recur frequently after resection but do not metastasize. Originally described as a tumor of the abdominal wall in women who had recently been pregnant, these rare, slow-growing tumors can arise at any site in the body. They have been classified according to anatomical location as extra-abdominal (60%), abdominal wall (25%), and intra-abdominal (15%) [1]. Abdominal desmoid tumour occurs sporadically or in association with some familial syndromes and often represents a clinical dilemma for surgeons. A wide range of clinical presentations that depend on location and vary from asymptomatic lesions to intestinal obstruction, bowel ischemia, functional deterioration of an ileal pouch-anal anastomosis, vascular or neural involvement, and urinary obstruction have been reported [2–5]. In this paper, we report an unusual presentation of an intra-abdominal desmoid tumour.

## 2. Case Report

A 17-year-old unmarried female patient presented with complaints of vague upper abdominal pain for the preceding six months with fullness of upper abdomen for the preceding one month. There was no history of jaundice, altered bowel habits, gastrointestinal bleeding, or abdominal trauma. Past medical or surgical history was unremarkable. There was no history of weight loss, loss of appetite, or oral contraceptive intake. No family history of similar ailment or malignancy was reported.

**Figure 1:**
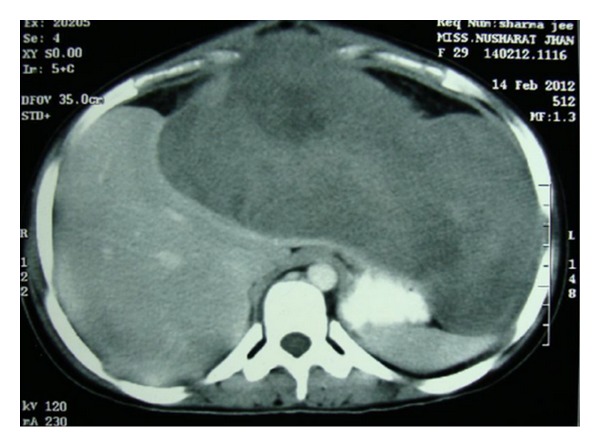
CECT abdomen showing the lesion.

**Figure 2:**
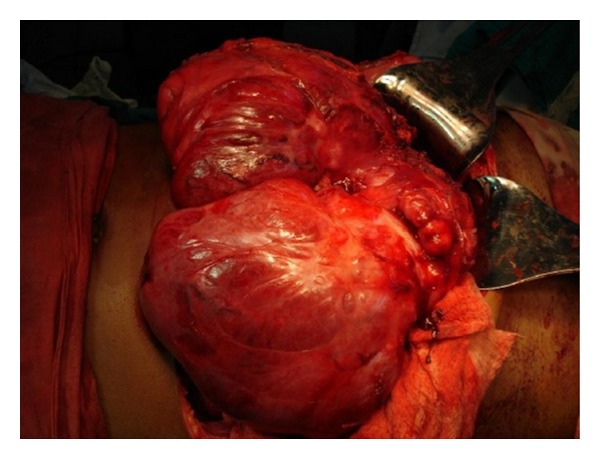
Intraoperative finding: bilobed, exophytic mass.

**Figure 3:**
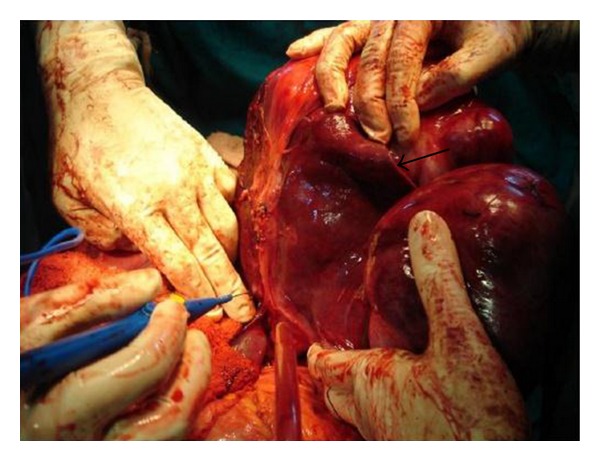
Relationship of mass with liver (black arrow indicates tumour-liver interface).

Abdominal examination revealed a firm, nontender, intraperitoneal mass (about 15 cm × 8 cm) with bosselated surface and rounded lower margin, which occupied the epigastrium and left hypochondrium. The lump moved with respiration. There was no organomegaly and no evidence of free fluid within the abdomen. An ultrasonography (USG) of the whole abdomen revealed a 14 cm × 10 cm mass arising from the left half of right lobe of liver with increased echogenicity suggestive of hepatoma. However, serum alpha fetoprotein (2.87 ng/mL) and liver function tests were normal. A contrast enhanced CT (CECT) scan of abdomen revealed a large, lobulated well-encapsulated hypodense, heterogeneously enhancing mass (178 mm × 99 mm × 182 mm) with an exophytic component arising from left lobe of liver, extending upto the gastrosplenic ligament, compressing the stomach and the spleen, and also abutting the anterior abdominal wall (Figure 1). CT-guided fine needle aspiration cytology suggested a diagnosis of a mesenchymal neoplasm with low/intermediate malignant potential. On the basis of the above findings, a left hepatectomy was planned. After proper preoperative preparation, the patient was explored. A large bilobed, exophytic mass (14 cm × 8 cm & 15 cm × 6 cm) arising from the superior surface of the left lobe of liver, abutting the right lobe, was found (Figures 2 and 3). It was most densely adherent to the left hemidiaphragm and part of central tendon. There was fibrosis near the region of suprahepatic inferior vena cava and no significant adherence of the mass with the anterior abdominal wall. No significant periportal lymphadenopathy was noted. A left hepatectomy was performed by finger fracture method, and the mass was excised with a portion of the left hemidiaphragm with which it was densely adherent. The postoperative period was uneventful, and the patient was discharged on postoperative day 7.

**Figure 4:**
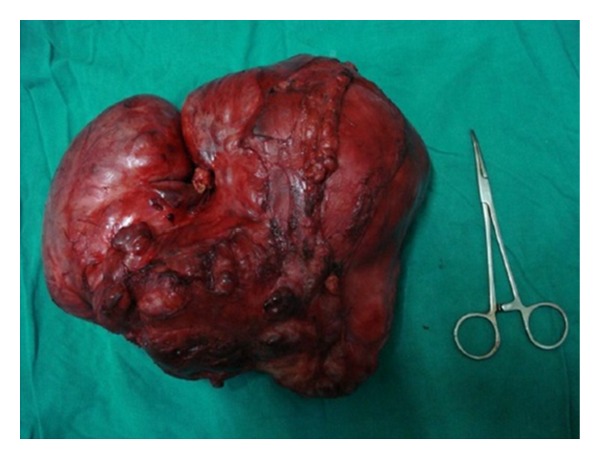
Resected specimen.

**Figure 5:**
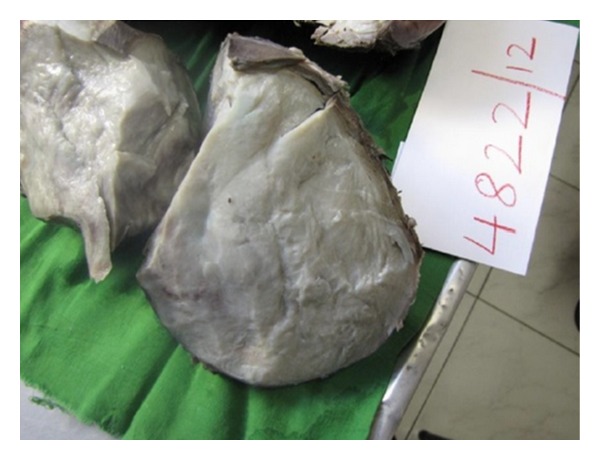
Fleshy cut section.

**Figure 6:**
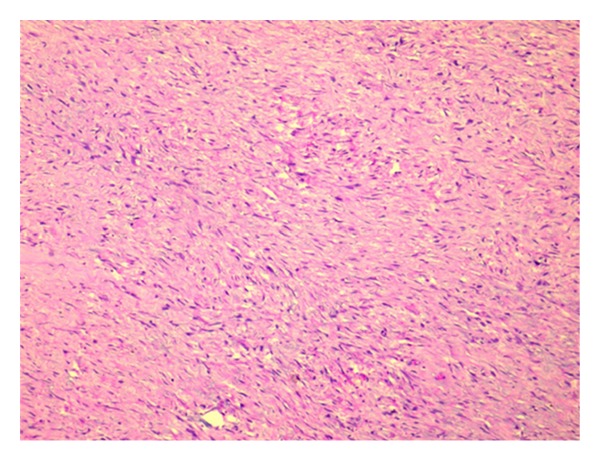
Photomicrograph of the tumour (10x H&E): elongated fascicles of spindle-shaped cells in a background of eosinophilic collagenized stroma.

**Figure 7:**
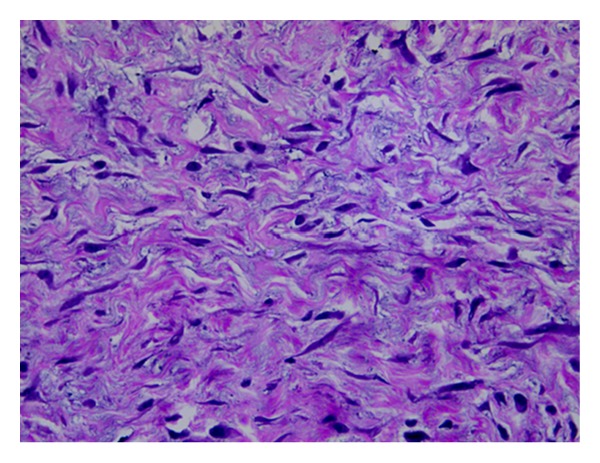
The individual tumour cells with pale eosinophilic cytoplasm, tapering vesicular nuclei, and inconspicuous nucleoli (40x H&E).

**Figure 8:**
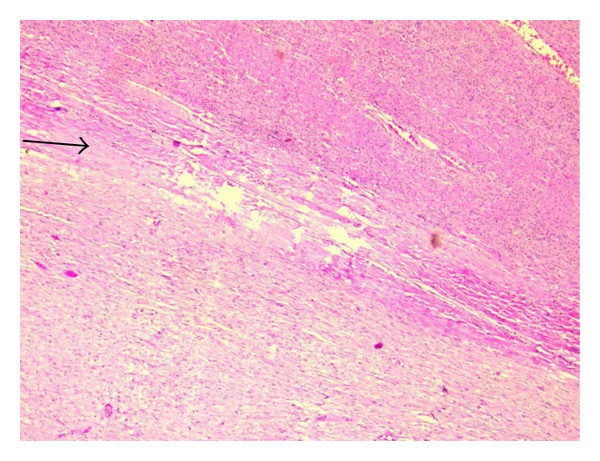
Photomicrograph showing fibrocollagenous septa (black arrow) separating the tumour from the adjacent liver parenchyma (10x H&E).

**Figure 9:**
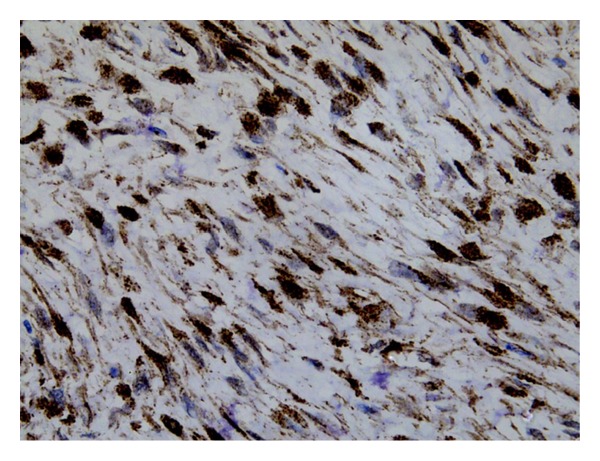
The cells showing immunoreactivity to beta-catenin (40x).

On macroscopic examination (Figure 4), the specimen was found to be bilobed, encapsulated, fleshy (Figure 5), reddish brown in colour with bosselated surface and a variegated consistency. Prominent vessels were seen on the surface. The specimen weighed 4.2 kg. Histopathology (Figures 6 and 7) showed that the tumour was composed of spindle-shaped cells with elongated nuclei lying in a myxoid background and interspersed with bands of collagen fibres. No nuclear pleomorphism or mitotic figures were seen. There were scanty inflammatory cells and plenty of fibroblasts, suggestive of benign spindle cell tumour. The tumour was well circumscribed and had a sharp capsular interface with the liver parenchyma (Figure 8). This feature ruled out its origin from the liver. Immunohistochemistry showed positivity of lesional fibroblastic cells for **β**-catenin (Figure 9) and negativity for CD34, CD117, EMA, SMA, desmin, vimentin, cytokeratin, and ALK1. A diagnosis of desmoid fibromatosis was suggested.

During followup, a colonoscopy was performed which did not reveal any colorectal polyps and hence excluded familial adenomatous polyposis (FAP). The patient is doing well for the last 6 months with no evidence of local recurrence on follow up USG abdomen.

## 3. Discussion

Desmoids account for about 0.03% of all neoplasms and <3% of all soft tissue tumors. Individuals in the age group of 15–60 years can be affected; desmoids are rare in the young and in the elderly [6–8], more common in women than in men [2], and have no significant racial or ethnic predilection.

Intra-abdominal desmoids are often observed in FAP (3.5–32%) and Gardner's syndrome or following abdominal trauma (surgical or nonsurgical) [6]. The association between antecedent trauma and the development of desmoids may be related to a possible molecular connection between wound-healing processes and fibroproliferative disorders of mesenchymal tissue [9]. Development of desmoids in patients taking oral contraceptives and its association with pregnancy have been reported [6].

 Intra-abdominal desmoids have been reported to arise from the retroperitoneum, pancreas, mesentery, and pelvis [7, 10–14]. Sporadic forms, which are more common, are defined as nontrauma or nongenetic related desmoids. They are generally extra-abdominal, most commonly involved areas are shoulder girdle, hip/buttock region, and the extremities, where the location is usually deep in the muscles or along fascial planes. Their biological behaviour and clinical course are different from their familial counterparts.

 There exist only a few reports in the literature on desmoids related to the diaphragm. Desmoid tumour of the posterior mediastinum, which has invaded into the diaphragm, has been reported [15, 16]. In another report a desmoid tumor involving the left anterior chest wall, upper abdomen, and diaphragm, which impinged on the left lung and displaced the liver, has been described. En bloc resection of this mass through a thoracoabdominal incision required resection of a part of the diaphragm and chest wall [17]. These reports suggest infiltration of the diaphragm by desmoid tumour. There is only one report in the literature on a diaphragmatic desmoid that is possibly primary [18] and no reference to an intra-abdominal desmoid masquerading as a hepatic mass.

 In the case we report, the tumour was most densely adherent to the under surface of diaphragm. The lesion appeared to arise from the left lobe of the liver on imaging (Figure 1) as well as on intraoperative evaluation (Figure 3). Histopathology, however, ruled out a hepatic origin based on a sharp capsular interface between the tumour and the adjacent liver tissue (Figure 8).

In both FAP and familial non-FAP tumors, mutations of the APC gene on the long arm of chromosome 5 have been incriminated. 85% of patients with desmoids were shown to have mutations in the beta-catenin gene. Somatic APC mutations as well as activating mutations of the beta-catenin gene have been discovered in the majority of sporadic desmoids. The resultant loss of ability to degrade beta-catenin and consequent elevation of beta-catenin levels promote fibroblastic proliferation through a nuclear mechanism [19]. Elucidation of the central role of beta-catenin in the pathogenesis of desmoid tumors may lead to future therapeutic advances [19–21].

Symptoms depend on the site of the tumor. Patients with intra-abdominal desmoid may have asymptomatic masses, symptoms of intestinal, vascular, and urinary obstruction, or neural involvement [3–5]. The diagnosis of desmoid is based on clinical suspicion. A personal or family history of FAP is frequently found.

Imaging in the form of CECT or Magnetic resonance imaging (MRI) is required to assess the degree of extension of the tumour to adjacent structures [6]. Incisional or core needle biopsy is performed for a histological diagnosis. Immunohistochemistry aids in confirming the diagnosis. Nuclear beta-catenin immunoreactivity supports the diagnosis of a desmoid tumor. In one large series [22] positive staining was identified in 80 and 67 percent of sporadic and FAP-associated desmoids, respectively. Beta-catenin negativity does not preclude the diagnosis of fibromatosis [9].

Desmoids are characterized by variable clinical behaviour. Although most grow progressively larger over time, growth is indolent. A multidisciplinary approach including surgery, chemotherapy, and radiation therapy is required for treatment.

## 4. Conclusion

Sporadic abdominal desmoids are rare tumours with varied presentations. We describe a desmoid tumour possibly arising from the diaphragm but presenting as a hepatic mass. This very rare case indicates that desmoid tumour should be included in the differential diagnosis of hepatic masses especially ones which are closely related to the diaphragm.
